# Comparison of weekly administration of cisplatin versus three courses of cisplatin 100 mg/m^2^ for definitive radiochemotherapy of locally advanced head-and-neck cancers

**DOI:** 10.1186/s12885-016-2478-8

**Published:** 2016-07-08

**Authors:** Dirk Rades, Daniel Seidl, Stefan Janssen, Amira Bajrovic, Katarina Karner, Primoz Strojan, Steven E Schild

**Affiliations:** Department of Radiation Oncology, University of Lubeck, Ratzeburger Allee 160, D-23538 Lübeck, Germany; Medical Practice for Radiotherapy and Radiation Oncology, Hannover, Germany; Department of Radiation Oncology, University Medical Center Eppendorf, Hamburg, Germany; Department of Radiotherapy, Institute of Oncology Ljubljana, Ljubljana, Slovenia; Department of Radiation Oncology, Mayo Clinic, Scottsdale, AZ USA

**Keywords:** Head-and-neck cancer, Definitive treatment, Radiochemotherapy, Cisplatin, Outcomes, Adverse events

## Abstract

**Background:**

To compare definitive radiochemotherapy with weekly administration of 30–40 mg/m^2^ of cisplatin to 100 mg/m^2^ of cisplatin on days 1, 22 and 43 for outcomes and toxicity in patients with squamous cell carcinoma of the head-and-neck.

**Methods:**

Seventy-five patients receiving radiochemotherapy with weekly cisplatin (30–40 mg/m^2^) were compared to 58 patients receiving radiochemotherapy with 100 mg/m^2^ cisplatin on days 1, 22 and 43. Radiochemotherapy regimen plus seven characteristics (age, gender, performance score, tumor site, T-/N-category, histologic grading) were evaluated for locoregional control (LRC), metastases-free survival (MFS) and overall survival (OS). Radiochemotherapy groups were compared for toxicity.

**Results:**

On multivariate analysis, improved LRC was associated with cisplatin 100 mg/m^2^ (hazard ratio [HR] 1.57; *p* = 0.008) and female gender (HR 4.37; *p* = 0.003). Radiochemotherapy regimen was not significantly associated with MFS on univariate analysis (*p* = 0.66). On multivariate analysis, better MFS was associated with ECOG performance score 0–1 (HR 5.63; *p* < 0.001) and histological grade 1–2 (HR 1.81; *p* = 0.002). On multivariate analysis, improved OS was associated with cisplatin 100 mg/m^2^ (HR 1.33; *p* = 0.023), ECOG performance score 0–1 (HR 2.15; *p* = 0.029) and female gender (HR 1.98; *p* = 0.026). Cisplatin 100 mg/m^2^ was associated with higher rates of grade ≥3 hematotoxicity (*p* = 0.004), grade ≥2 renal failure (*p* = 0.004) and pneumonia/sepsis (*p* = 0.033).

**Conclusions:**

Radiochemotherapy with 100 mg/m^2^ of cisplatin every 3 weeks resulted in better LRC and OS than weekly doses of 30–40 mg/m^2^. Given the limitations of a retrospective study, 100 mg/m^2^ of cisplatin appears preferable. Since this regimen was associated with considerable acute toxicity, patients require close monitoring.

## Background

Many patients with locally advanced squamous cell carcinoma of the head-and-neck (SCCHN) are not candidates for surgical resection and receive definitive radiotherapy. After randomized trials had demonstrated that radiochemotherapy was superior to radiotherapy alone for definitive treatment of SCCHN, radiochemotherapy became the standard treatment for these patients [1–3]. According to a large meta-analysis, concurrent administration of radiochemotherapy resulted in significantly better outcomes than sequential approaches [4]. This meta-analysis included patients who received radiochemotherapy with cisplatin alone or various poly-chemotherapy regimens, including combined cisplatin-based regimens, but did not show significantly superiority of a particular regimen. Thus, the most appropriate chemotherapy given concurrently with radiation therapy for locally advanced SCCHN requires further clarification.

In two randomized trials comparing radiochemotherapy and radiotherapy alone after surgery for SCCHN in patients with risk factors, radiochemotherapy with 100 mg/m^2^ of concurrent cisplatin given on days 1, 22 and 43 was significantly superior to radiotherapy alone with respect to treatment outcomes [5, 6]. In definitive radiotherapy setting, the same cisplatin regimen was also tested in phase III randomized fashion [7, 8]. Guided by these trials, radiochemotherapy with three courses 100 mg/m^2^ of cisplatin became the preferred regimen for both definitive and postoperative in many institutions. However, other centers are concerned about this regimen, since it was reported to be very toxic [9]. Therefore, other cisplatin-regimens have been introduced to the radiochemotherapy of SCCHN. One of these alternative regimens is weekly administration of 30–40 mg/m^2^ of cisplatin. In 2008, a retrospective study showed that weekly administration of 33–40 mg/m^2^ of cisplatin was better tolerated than 80–100 mg/m^2^ of cisplatin given every 3 weeks [10].

Three retrospective studies and one randomized study of 50 eligible patients had compared higher-dose cisplatin (100 mg/m^2^ on days 1, 22 and 43) to weekly administration of 30 or 40 mg/m^2^ of cisplatin for non-nasopharyngeal SCCHN [11–14]. However, these studies produced inconsistent results with respect to treatment outcomes. One retrospective study suggested that 100 mg/m^2^ of cisplatin resulted in better overall survival (OS) and similar progression-free survival (PFS) compared to weekly cisplatin [11]. In another retrospective study of patients receiving definitive (30 %) or adjuvant (70 %) radiochemotherapy, 100 mg/m^2^ of cisplatin resulted in significantly better PFS and OS on univariate analyses but not on multivariate analyses [12]. In the other two studies, outcomes were not significantly different with 100 mg/m^2^ of cisplatin given every 3 weeks or weekly administration of cisplatin [13, 14]. Of the latter two studies, the small prospective trial was limited to patients with cancer of the oral cavity, and the retrospective study was performed in patients receiving postoperative radiochemotherapy (*N* = 104). Taking into account the available data from the literature, it becomes obvious that more studies comparing 100 mg/m^2^ of cisplatin every 3 weeks to weekly administration of 30 or 40 mg/m^2^ are required, particularly in patients receiving definitive radiochemotherapy for SCCHN. Therefore, the present study included only SCCHN patients receiving definitive radiochemotherapy. It aimed to contribute to the question whether weekly cisplatin is a reasonable and less toxic alternative to 100 mg/m^2^ of cisplatin given every 3 weeks.

## Methods

A total of 133 patients treated with definitive radiochemotherapy for histologically confirmed locally advanced unresectable SCCHN between 2003 and 2014 were included in this retrospective study, which was approved by the local ethics committee (University of Lübeck). Seventy-five patients had received weekly cisplatin doses of 30–40 mg/m^2^ and were compared to 58 patients treated with 100 mg/m^2^ of cisplatin given on days 1, 22 and 43. Patients receiving Cisplatin weekly were mainly from Ljubljana, and those receiving100 mg/m2 of cisplatin on days 1, 22 and 43 were mainly from Northern Germany. Chemotherapy regimens were selected according to interdisciplinary treatment protocols preferred at the contributing institutions at the time the patients were treated. Both groups were not significantly different regarding the distribution of patient characteristics including age, gender, Eastern Cooperative Oncology Group (ECOG) performance score, primary tumor site, T-category, N-category, histologic grading and cumulative cisplatin dose (Table 1). Cancer of the oral cavity was also included in this study although response to radiochemotherapy is often not satisfactory for these tumors, since it represents a common site of SCCHN. The proportion of patients with cancer of the oral cavity was similar in both groups (11 versus 12 %, Table 1).

**Table 1:** Comparison of the distributions of patient characteristics in the radiochemotherapy groups (30–40 mg/m2 of cisplatin weekly vs. 100 mg/m^2^ of cisplatin on days 1, 22 and 43; Chi-square test)

	Cisplatin weekly *N* patients (%)	Cisplatin 100 mg/m^2^ *N* patients (%)	*P*
Age			
≤56 years (*N* = 67)	37 (49)	30 (52)	
≥ 57 years (*N* = 66)	38 (51)	28 (48)	0.92
Gender			
Female (*N* = 29)	15 (20)	14 (24)	
Male (*N* = 104)	60 (80)	44 (76)	0.86
ECOG Performance score			
0–1 (*N* = 115)	64 (85)	51 (88)	
2 (*N* = 18)	11 (15)	7 (12)	0.92
Primary tumor site			
Oropharynx (*N* = 69)	36 (48)	33 (57)	
Hypopharynx (*N* = 19)	12 (16)	7 (12)	
Larynx (*N* = 30)	19 (25)	11 (19)	
Oral cavity/Floor of mouth (*N* = 15)	8 (11)	7 (12)	0.88
T-category			
T1-2 (*N* = 16)	9 (12)	7 (12)	
T3-4 (*N* = 117)	66 (88)	51 (88)	0.99
N-category			
N0-2a (*N* = 66)	39 (52)	27 (47)	
N2b-3 (*N* = 67)	36 (48)	31 (53)	0.75
Histologic grading			
G 1–2 (*N* = 85)	49 (65)	36 (62)	
G3 (*N* = 48)	26 (35)	22 (38)	0.89
Cumulative cisplatin dose			
≤200 mg/m^2^ (*N* = 85)	51 (68)	34 (59)	
>200 mg/m^2^ (*N* = 48)	24 (32)	24 (41)	0.50

After Bonferroni correction for multiple tests (8 tests), *p*-values of <0.006 were considered significant

Definitive radiotherapy was performed with 6–10 MV photon beams from a linear accelerator as three-dimensional conformal radiotherapy after computed tomography-based treatment planning. Patients treated with intensity modulated radiotherapy (IMRT) and volumetric modulated arc therapy (VMAT) were not included. The planned total radiation dose administered to the primary tumor and the involved lymph nodes was 70 Gy given in 2-Gy fractions on 5 days per week (conventional fractionation). Total doses to lymph nodes were 50–60 Gy. Concurrent cisplatin was given as bolus infusion of 30–40 mg/m^2^ once a week or as bolus infusion of 100 mg/m^2^ on days 1, 22 and 43. All patients received prophylactic hydration and antiemetic agents and were monitored for potential toxicity (clinical examination, blood samples) at least weekly.

The radiochemotherapy regimen and eight additional characteristics (Table 1) were evaluated with respect to LRC, MFS and OS. The HPV-status was available only in a few patients and, therefore, not analyzed. Radiochemotherapy regimens were additionally compared for acute and late adverse events (Common Terminology Criteria of Adverse Events (CTCAE) version 4.0) [15]. The follow-up schedule included visits every 3 months for 2 years, every 6 months during the third year, and every 12 months thereafter. Additional visits were performed when toxicity-related symptoms occurred or progressive disease was suspected.

LRC, MFS and OS were referenced form the last day of radiotherapy and calculated with the Kaplan-Meier-method [16]. The corresponding Kaplan-Meier curves were compared using the log-rank test. Those characteristics found to be significant (*p* < 0.006 after Bonferroni correction for multiple tests representing an alpha level of 0.05) or showed a trend (*p* < 0.055) on univariate analyses were subsequently analyzed in a multivariate manner with the Cox proportional hazards model. In the multivariate analyses, *p*-values of <0.05 were considered significant. For comparisons of the radiochemotherapy groups for acute and late adverse events, the Chi-square test was used.

## Results

Median follow up times were 21 months (range: 0–80 months) in the entire cohort and 38 months (range: 4–80 months) in those patients being alive at their last follow up visit.

**Fig. 1:**
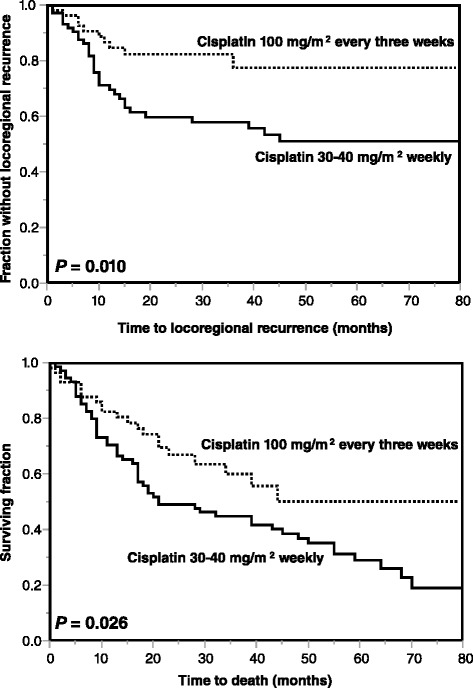
Comparison of the radiochemotherapy groups (30–40 mg/m^2^ of cisplatin weekly vs. 100 mg/m^2^ of cisplatin every 3 weeks) for locoregional control (*top*) and overall survival (*bottom*)

On univariate analyses, cisplatin 100 mg/m^2^ (Fig. 1, *p* = 0.010), female gender (*p* = 0.010) and favorable (oropharynx or larynx) primary tumor site (*p* = 0.047) showed a trend towards improved LRC (Table 2). In the multivariate analysis of LRC, radiochemotherapy regimen (*p* = 0.008) and gender (*p* = 0.003) were significant, whereas primary tumor site (*p* = 0.16) did not achieve significance (Table 5).

**Table 2:** Univariate analysis of locoregional control (LRC)

	At 1 year (%)	At 3 years (%)	*P*
Radiochemotherapy regimen			
Cisplatin weekly (*N* = 75)	70	58	
Cisplatin 100 mg/m^2^ (*N* = 58)	85	78	0.010
Age			
≤56 years (*N* = 67)	78	65	
≥57 years (*N* = 66)	74	68	0.72
Gender			
Female (*N* = 29)	92	87	
Male (*N* = 104)	72	61	0.010
ECOG Performance score			
0–1 (*N* = 115)	78	69	
2 (*N* = 18)	66	49	0.14
Primary tumor site			
Oropharynx (*N* = 69)	83	74	
Hypopharynx (*N* = 19)	47	47	
Larynx (*N* = 30)	79	67	
Oral cavity/Floor of mouth (*N* = 15)	75	56	0.047
T-category			
T1–2 (*N* = 16)	93	76	
T3–4 (*N* = 117)	74	65	0.28
N-category			
N0-2a (*N* = 66)	76	68	
N2b-3 (*N* = 67)	76	65	0.93
Histologic grading			
G 1–2 (*N* = 85)	77	69	
G3 (*N* = 48)	75	63	0.65
Cumulative cisplatin dose			
≤200 mg/m^2^ (*N* = 85)	70	62	
>200 mg/m^2^ (*N* = 48)	86	74	0.09

After Bonferroni correction for multiple tests, *p*-values of <0.006 were considered significant

In the entire cohort, MFS rates at 1 and 3 years were 86 and 71 %, respectively. On univariate analysis, improved MFS was associated with ECOG performance score 0–1 (*p* < 0.001), favorable (oropharynx or larynx) primary tumor site (*p* = 0.002), N-category 0–2a (*p* = 0.001) and histological grade 1–2 (*p* = 0.003) (Table 3). The radiochemotherapy regimen was not significantly associated with MFS (*p* = 0.66). On multivariate analysis of MFS, ECOG performance score (*p* < 0.001) and histological grading (*p* = 0.002) achieved significance, whereas N-category (*p* = 0.09) and primary tumor site (*p* = 0.30) were not significant (Table 5).

**Table 3:** Univariate analysis of metastases-free survival (MFS)

	At 1 year (%)	At 3 years (%)	*P*
Radiochemotherapy regimen			
Cisplatin weekly (*N* = 75)	89	68	
Cisplatin 100 mg/m^2^ (*N* = 58)	83	76	0.66
Age			
≤56 years (*N* = 67)	85	71	
≥57 years (*N* = 66)	88	72	0.30
Gender			
Female (*N* = 29)	79	69	
Male (*N* = 104)	89	72	0.59
ECOG Performance score			
0–1 (*N* = 115)	92	79	
2 (*N* = 18)	52	25	<**0.001**
Primary tumor site			
Oropharynx (*N* = 69)	89	78	
Hypopharynx (*N* = 19)	71	61	
Larynx (*N* = 30)	100	77	
Oral cavity/Floor of mouth (*N* = 15)	62	44	**0.002**
T-category			
T1–2 (*N* = 16)	93	85	
T3–4 (*N* = 117)	85	69	0.93
N-category			
N0-2a (*N* = 66)	97	81	
N2b-3 (*N* = 67)	76	62	**0.001**
Histologic grading			
G 1–2 (*N* = 85)	95	76	
G3 (*N* = 48)	72	63	**0.003**
Cumulative cisplatin dose			
≤200 mg/m^2^ (*N* = 85)	87	71	
>200 mg/m^2^ (*N* = 48)	86	73	0.69

After Bonferroni correction for multiple tests, *p*-values of <0.006 were considered significant

Bold values represent significant *p*-values

In the entire cohort, median survival time was 39 months, and the OS rates at 1 and 3 years were 76 and 51 %, respectively. In the univariate analyses, better OS was significantly associated with favorable (oropharynx or larynx) primary tumor site (*p* < 0.001). Cisplatin 100 mg/m^2^ (Fig. 1, *p* = 0.024), ECOG performance score 0–1 (*p* = 0.006) and female gender showed a trend (*p* = 0.050) (Table 4). On multivariate analysis of OS, radiochemotherapy regimen (*p* = 0.023), ECOG performance score (*p* = 0.029) and gender (*p* = 0.026) achieved significance, whereas primary tumor site (*p* = 0.32) did not (Table 5).

**Table 4:** Univariate analysis of overall survival (OS)

	At 1 year (%)	At 3 years (%)	*P*
Radiochemotherapy regimen			
Cisplatin weekly (*N* = 75)	71	45	
Cisplatin 100 mg/m^2^ (*N* = 58)	83	60	0.026
Age			
≤56 years (*N* = 67)	77	54	
≥57 years (*N* = 66)	74	49	0.50
Gender			
Female (*N* = 29)	89	70	
Male (*N* = 104)	72	47	0.050
ECOG Performance score			
0–1 (*N* = 115)	78	56	
2 (*N* = 18)	61	14	0.006
Primary tumor site			
Oropharynx (*N* = 69)	78	61	
Hypopharynx (*N* = 19)	53	21	
Larynx (*N* = 30)	83	59	
Oral cavity/Floor of mouth (*N* = 15)	80	29	<**0.001**
T-category			
T1–2 (*N* = 16)	81	63	
T3–4 (*N* = 117)	75	50	0.85
N-category			
N0-2a (*N* = 66)	79	60	
N2b-3 (*N* = 67)	73	42	0.14
Histologic grading			
G 1–2 (*N* = 85)	78	52	
G3 (*N* = 48)	73	51	0.47
Cumulative cisplatin dose			
≤ 200 mg/m^2^ (*N* = 85)	69	46	
> 200 mg/m^2^ (*N* = 48)	87	61	0.13

After Bonferroni correction for multiple tests, *p*-values of <0.006 were considered significant

Bold values represent significant *p*-values

**Table 5:** Results of the multivariate analyses of locoregional control, metastases-free survival and overall survival

	Hazard ratio	95 %-confidence interval	*P*
Locoregional control			
Radiochemotherapy regimen			
(Cisplatin 100 mg/m2 vs. Cisplatin weekly)	1.57	1.12–2.31	**0.008**
Gender			
(female vs. male)	4.37	1.58–18.11	**0.003**
Primary tumor site			
(oropharynx or larynx vs. others)	1.18	0.94–1.45	0.16
Metastases-free survival			
ECOG performance score			
(0–1 vs. 2)	5.63	2.19–14.11	<**0.001**
N-category			
(N0-2a vs. N2b-3)	2.02	0.90–4.84	0.09
Histological grading			
(G1–2 vs. G3)	1.81	1.26–2.66	**0.002**
Primary tumor site			
(oropharynx or larynx vs. others)	1.15	0.88–1.50	0.30
Overall survival			
Radiochemotherapy regimen			
(Cisplatin 100 mg/m2 vs. Cisplatin weekly)	1.33	1.04–1.73	**0.023**
Gender			
(female vs. male)	1.98	1.08–3.96	**0.026**
ECOG performance score			
(0–1 vs. 2)	2.15	1.09–3.99	**0.029**
Primary tumor site			
(oropharynx or larynx vs. others)	1.09	0.92–1.30	0.32

Bold values represent significant *p*-values

The comparison of both radiotherapy groups for acute and late adverse events revealed that 100 mg/m^2^ of cisplatin was associated with significantly higher rates of grade ≥3 hematotoxicity (*p* = 0.004), grade ≥2 renal failure (*p* = 0.004), and pneumonia/sepsis showed a trend (*p* = 0.033) (Table 6). The rates of grade ≥2 oral mucositis (*p* = 0.95), grade ≥2 skin toxicity (*p* = 0.25), grade ≥2 xerostomia (*p* = 0.44) and grade ≥2 subcutaneous fibrosis (*p* = 0.20) were not significantly different in both groups. The complete planned chemotherapy could be administered in 63 % (47/75) of patients in the cisplatin weekly group and in 50 % (29/58) of patients in the cisplatin 100 mg/m^2^ group, respectively (*p* = 0.34). A total radiation dose of 70 Gy could be administered in 92 % (69/75) and 95 % (55/58) of patients, respectively (*p* = 0.87). Death during radio-chemotherapy occurred in 4 % (3/75) and 2 % (1/58) of patients, respectively (*p* = 0.48).

**Table 6:** Comparison of the radiochemotherapy groups (30–40 mg/m2 of cisplatin weekly vs. 100 mg/m^2^ of cisplatin on days 1, 22 and 43) for acute and late adverse events

	Cisplatin weekly *N* patients (%)	Cisplatin 100 mg/m^2^ *N* patients (%)	*P*
Oral mucositis			
Grade ≥2	70 (93)	55 (95)	0.95
Skin reactions			
Grade ≥2	48 (64)	48 (83)	0.25
Hematotoxicity			
Grade ≥3	7 (9)	19 (33)	**0.004**
Renal failure			
Grade ≥2	2 (3)	12 (21)	**0.004**
Pneumonia/Sepsis			
Grade ≥3	1 (1)	7 (12)	0.033
Xerostomia^a^			
Grade ≥2	28/60 (47)	34/58 (59)	0.44
Subcutaneous fibrosis^a^			
Grade ≥2	27/72 (38)	28/51 (55)	0.20

^a^not available in all patients

After Bonferroni correction for multiple tests (7 tests), *p*-values of <0.007 were considered significant

Bold values represent significant *p*-values

## Discussion

Definitive radiochemotherapy is one of the most common treatment approaches for locally advanced SCCHN. In order to achieve the best possible outcomes, irradiation and chemotherapy should be administered concurrently [4]. The most important agent for definitive radiochemotherapy of SCCHN is cisplatin either given alone or as part of combined chemotherapy regimens. The most commonly used of these regimens worldwide is 100 mg/m^2^ of cisplatin alone given every 3 weeks, i.e. on days 1, 22 and 43. This regimen can be associated with high rates of severe adverse events [9]. Therefore, alternative cisplatin regimens became relatively popular for radiochemotherapy of SCCHN, such as two courses of 20 mg/m^2^ cisplatin on five consecutive days or weekly administration of 30–40 mg/m^2^ [10–14, 17, 18]. The latter regimen is particularly used for patients who do not wish to stay in hospital during chemotherapy.

It is not yet clear whether weekly administration of 30–40 mg/m^2^ cisplatin is as effective as the “standard” regimen 100 mg/m^2^ of cisplatin given every 3 weeks. The available studies performed in patients with non-nasopharyngeal SCCHN produced inconsistent results. In a retrospective study of 94 patients, 100 mg/m^2^ of cisplatin resulted in better OS (*p* = 0.041) and similar PFS (*p* = 0.47) [11]. However, patients in the cisplatin-weekly group were significantly older (*p* = 0.001), which likely have introduced a bias. A more recent retrospective study suggested that 100 mg/m^2^ cisplatin every 3 weeks resulted in better PFS and OS than weekly administration of 40 mg/m^2^ cisplatin [12]. The 5-year PFS rates were 56 and 44 %, respectively, and the 5-year OS rates 62 and 53 %, respectively. Both differences achieved significance in the univariate analyses but not in the multivariate analyses. In that study, 30 % of patients received definitive radiochemotherapy and 70 % radiochemotherapy following surgery or induction chemotherapy. The heterogeneity of treatment regimens may have confounded the results. Another retrospective study compared 100 mg/m^2^ cisplatin every 3 weeks to weekly administration of 30 mg/m^2^ cisplatin in a more homogeneously treated cohort of patients, who all received radiochemotherapy following surgery [13]. Three-year LRC rates were 71 and 74 %, respectively (*p* = 0.95), and 3-year OS rates 84 and 75 %, respectively (*p* = 0.30). In addition to these retrospective studies, one randomized trial was performed that compared 100 mg/m^2^ cisplatin every 3 weeks to 40 mg/m^2^ cisplatin weekly [14]. The 1-year LRC rates were 71 and 60 %, respectively (*p* = 0.81), and 1-year OS rates were 79 and 72 %, respectively (*p* = 0.98). The sample size of 50 eligible patients was too small to achieve an adequate statistical power. Furthermore, the trial was limited to patients with cancer of the oral cavity and may not be generalized to other sites of SCCHN.

Thus, more studies comparing 30–40 mg/m^2^ weekly to 100 mg/m^2^ given every 3 weeks for radiochemotherapy of SCCHN would be helpful, ideally in form of a randomized trial with an adequate statistical power. However, such a trial will likely be difficult to perform, since most centers wish to keep on using their preferred radiochemotherapy regimen. Therefore, the present retrospective study was initiated to provide additional information to answer this important question. It included only patients, who had received definitive radiochemotherapy, to avoid a potential selection bias caused by different types of treatment. However, when interpreting the results of this study one has to keep in mind that this study is retrospective in nature. Retrospective studies always bear the risk of including hidden selection biases.

There could have been different proportions of HPV-positive tumors in both radiochemotherapy groups. The HPV-status was not available in most patients and, therefore, not included in the analyses. In previous reports from Slovenia and Northern Germany, 20 and 15 % respectively of oropharynx cancers were HPV-positive [19, 20]. Further limitations of this study included the facts that the radiochemotherapy groups were not compared for treating institution, that patients receiving IMRT or VMAT were not included and that both radiochemotherapy groups were compared for patient characteristics with the Chi-square test instead of using propensity score matching.

According to the results of the present study, 100 mg/m^2^ cisplatin given every 3 weeks led to better LRC and OS than weekly administration of 30–40 mg/m^2^ cisplatin. Summarizing the results of both studies with respect to treatment outcomes, 100 mg/m^2^ cisplatin appears preferable to weekly administration of 30–40 mg/m^2^ cisplatin for definitive radiochemotherapy of SCCHN. However, one question is whether improved outcomes are impaired by more serious adverse events? Ho et al. reported that 100 mg/m^2^ cisplatin was less tolerated than weekly administration of 40 mg/m^2^ cisplatin [10]. In contrast, *Tsan* et al. observed a higher rate of grade ≥3 oral mucositis (75 versus 39 %, *p* = 0.012) and a higher rate of grade ≥3 overall toxicity (92 versus 81 %, *p* = 0.02) in the 40 mg/m^2^ cisplatin-weekly group [14]. In the study of *Espeli* et al., 100 mg/m^2^ cisplatin resulted in more renal failures (*p* = 0.04) [11]. In the largest study so far (Fayette et al.), 100 mg/m^2^ cisplatin was associated with significantly more adverse events than weekly administration of 40 mg/m^2^ cisplatin [12]. The rates of grade 3/4 mucositis were 34 and 12 %, respectively (*p* < 0.001), and the rates of grade 3/4 dermatitis were 7 and 1 %, respectively (*p* = 0.014). Decrease of creatinine clearance was also more pronounced in the 100 mg/m^2^ cisplatin group (*p* < 0.001). Also in the present study, some of the acute adverse events were significantly more frequent in the 100 mg/m^2^ cisplatin group (Table 5). These findings demonstrate that patients receiving definitive radiochemotherapy with 100 mg/m^2^ cisplatin on days 1, 22 and 42 require intensive monitoring (clinical examination, bone marrow function, renal function) and timely supportive care. If they are able to withstand this intensive radiochemotherapy regimen, they can benefit in terms of LRC and OS. I may be questioned why more patients treated with 100 mg/m^2^ cisplatin received a cumulative dose >200 mg/m^2^ than in the weekly cisplatin group, although 100 mg/m^2^ cisplatin was associated with more acute toxicity? This finding can to a certain extent be explained by the reduced compliance of some patients. In the cisplatin weekly group, the weekly cisplatin dose was 30 mg/m^2^ in 71 of 75 patients. If such a patient refused the last administration of cisplatin, the cumulative dose was only 180 mg/m^2^. Of the 71 patients receiving weekly cisplatin doses of 30 mg/m^2^, nine patients (13 %) received a cumulative dose of only 180 mg/m^2^ without developing a grade 3 acute toxicity.

## Conclusions

Definitive radiochemotherapy with 100 mg/m^2^ of cisplatin given on days 1, 22 and 43 resulted in better LRC and OS than weekly doses of 30–40 mg/m^2^. Thus, 100 mg/m^2^ of cisplatin appears preferable for definitive radiochemotherapy of locally advanced SCCHN. However, one should be aware that the regimen including 100 mg/m^2^ of cisplatin given every 3 weeks is associated with considerable acute toxicity. Patients receiving this regimen need close monitoring and timely supportive care.

## Abbreviations

CI, confidence interval; ECOG, Eastern Cooperative Oncology Group; Gy, gray; HPV, human papilloma virus; HR, hazard ratio; LRC, locoregional control; MFS, metastases-free survival; OS, overall survival; PFS, progression.free survival; SCCHN, squamous cell carcinoma of the head-and-neck
